# Brushite-Forming Mg-, Zn- and Sr-Substituted Bone Cements for Clinical Applications

**DOI:** 10.3390/ma3010519

**Published:** 2010-01-18

**Authors:** Sandra Pina, José M.F. Ferreira

**Affiliations:** Department of Ceramics and Glass Engineering, University of Aveiro, CICECO, 3810-193 Aveiro, Portugal; E-Mail: jmf@ua.pt (J.M.F.F.)

**Keywords:** brushite, magnesium, strontium, zinc, bone cements

## Abstract

Calcium phosphate cements have been in clinical use for the last 10 years. Their most salient features include good biocompatibility, excellent bioactivity, self-setting characteristics, low setting temperature, adequate stiffness, and easy shaping to accomodate any complicated geometry. They are commonly used in filling bone defects and trauma surgeries as mouldable paste-like bone substitute materials. Substitution of trace elements, such as Mg, Sr and Zn ions, into the structure of calcium phosphates is the subject of widespread investigation nowadays, because of their impending role in the biological process. Subtle differences in composition and structure of these materials may have a profound effect on their *in vivo* behaviour. Therefore, the main goal of this paper is to provide a simple, but comprehensive overview of the present achievements relating to brushite-forming cements doped with Mg, Zn and Sr, and to identify new developments and trends. In particular, the influence of ionic substitution on the chemical, physical and biological properties of these materials is discussed.

## 1. Introduction

Bone substitutes should have a good local and systemic compatibility, the capability of being substituted by bone and of completely filling any defect. These features require osteoconductive and/or osteoinductive properties of the implant comparable to those of the natural bone.

Currently available bone substitutes show a variety of compositions and properties. Among them, compounds made of inorganic calcium phosphates (CaP) are frequently used. They are not toxic and do not cause cell death in the surrounding tissues. The biological response to these materials follows a similar cascade observed in fracture healing. This cascade includes hematoma formation, inflammation, neovascularisation, osteoclastic resorption, and new bone formation. CaP undergo processes of dissolution and precipitation resulting in a strong material-bone interface [1,2,3].

The first clinical attempt to use CaP compounds was reported in 1920 by Albee, in the repair of a bony defect [4]. A second clinical report was only published 30 years later [5]. Levitt *et al.* [6] and Monroe *et al.* [7] also suggested CaP ceramic material for bone and tooth implants. Between 1976 and 1986, serious efforts were made toward development and commercialization of CaP as biomaterials for bone repair, substitution and augmentation [8,9,10,11]. Later, tricalcium phosphate (TCP) was used to repair surgically-created infrabony defects in dogs [12] and for alveolar ridge augmentation [13], and dense hydroxyapatite (HA) cylinders were used as dental root implants after tooth extraction [14,15].

In the past two decades, CaP biomaterials have gained acceptance in dental and orthopaedic applications, such as, repair of bone defects, tooth root replacements, ear implants, spine fusion, and coatings on orthopaedic and dental implants [8,13,15,16,17,18]. CaP-based cements that harden inside bone defects are other usually used bone grafts [16,19,20,21].

At present, there are two types of CaP cements (CPCs) depending on the end-product of the reaction: apatite (AP) cements and dicalcium phosphate dihydrate (DCPD or brushite) cements [22,23]. Apatite is formed above pH 4.2, while brushite is preferentially formed in calcium phosphate cements when the pH value of the paste is < 4.2 [24], although it may grow even up to pH 6.5. Brushite cements have raised special interest because they are resorbed *in vivo* much faster than apatite ones [25,26]. Moreover, brushite is metastable under physiological conditions and brushite based cements possess shorter setting times [24].

In recent years, ionic incorporations in CaP (namely, α, β-TCP) ceramics, such as magnesium (Mg), strontium (Sr) and zinc (Zn), have been the subject of great interest owing to the critical role of these ions in the biological processes after implantation [27,28,29,30,31,32,33,34,35]. Mg has its own significance in the calcification process and on bone fragility and has indirect influence on mineral metabolism [27]; it also has influence on HA crystal formation and growth [28,29,30]. Furthermore, a deficiency of Mg in bone has been suggested as a possible risk factor for osteoporosis in humans [28,29]. Zn is an essential trace element for promoting osteoblastic cell proliferation and differentiation and thought to possess a potent and selective inhibitory effect on osteoclastic bone resorption *in vivo* [33,34]. The Zn^2+^ ion is involved in many metallo-enzymes and proteins, including alkaline phosphatase (ALP). Sr has beneficial effects in the treatment of osteoporosis due to the prevention of bone loss by a mechanism of depressing bone resorption and maintaining bone formation and can be deposited into the mineral structure of bone, especially in regions of high metabolic turnover [35,36,37]. In addition, Sr increases osteoclast apoptosis and enhances preosteoblastic cell proliferation and collagen synthesis.

The goal of the present paper is to provide the reader with a brief overview of the present achievements relating to brushite-forming cements doped with Mg, Zn and Sr, and to identify the newest developments and trends. In particular, the influence of ionic substitutions on the chemical, physical and biological properties of these materials will be discussed.

## 2. Calcium Phosphate Cements

In 1832, Ostermann prepared a CaP biomaterial in the form of a paste that set *in situ* to form a solid material [38]. Nevertheless, Brown and Chow in 1986 [20] were the first to present this new form of CaPs, currently known as calcium phosphate cements (CPCs). CPCs are resorbable, promote development of osteoconductive pathways, possess sufficient compressive strength for a number of applications, are noncytotoxic, create chemical bonds to the host bones, restore contour and have both chemical compositions and X-ray diffraction patterns similar to those of bone [39,40,41,42]. The major advantages of the CPCs include a fast setting time, excellent mouldability, outstanding biocompatibility, and easy manipulation [40,43]; therefore, the cements are more versatile in handling characteristics than prefabricated CaP granules or blocks. Besides, like any other bioceramics, CPCs provide the opportunity for bone grafting using alloplastic materials, which are unlimited in quantity and provide no risk of infectious diseases [42]. As a matter of fact, one of the main factors of grafting failure with bone substitutes is infection and the risk of infection is proportional to the amount of bone graft. 

CPCs are made of an aqueous solution and of one or several CaPs, which upon mixing, dissolve and precipitate into a less soluble CaP and set by the entanglement by the growth of crystals, providing a mechanical rigidity to the cement. Then, the paste can be placed into a defect as a substitute for the damaged part of bone, where it hardens *in situ* within the operating theatre. It hardens in generally < 20 min at body temperature (37 °C) and then displays limited solubility. The relative stability and solubility of various CaPs is the major driving force for the setting reactions that occur in CPCs, being therefore dependent upon the pH value of a cement paste. The formation of brushite (or dicalcium phosphate dihydrate, DCPD) cements requires acidic pH values, while apatite (AP) cements are formed under neutral-alkaline conditions [24].

### 2.1. Brushite Cements

Brushite cements were introduced in 1987 by Mirtchi and Lemaitre, where DCPD is the major end product of the setting reaction between β-TCP and monocalcium phosphate monohydrate (MCPM), as expressed by equation (1):

β − Ca_3_(PO_4_)_2_ + Ca(H_2_PO_4_)_2_H_2_O + 7H_2_O → 4CaHPO_4_2H_2_O (1)

Other formulations have been already proposed, such as β-TCP + H_3_PO_4_ and TTCP + MCPM + CaO. According to Bohner *et al.* [44], the β-TCP + H_3_PO_4_ formulations have several advantages over β-TCP + MCPM formulations, namely: (i) easier and faster preparation, (ii) a better control of the chemical composition and reactivity, and (iii) improved physico-chemical properties, such as longer setting times and larger tensile strengths due to a higher homogeneity. However, they also state that the use of H_3_PO_4_ might weaken the biocompatibility of the cement formulation, owing to low pH values during setting [44].

Brushite cements are acidic during setting, since DCPD can only precipitate from solutions at pH values below 6; hence the reaction is very rapid corresponding to the initial setting stage. Despite this initial high reactivity, the hardening stage of brushite cements typically last one day until completion, due to the increasing of the paste pH at the end of the setting reaction. In order to control the start of the setting reaction, inhibitors of crystal nucleation and growth, and less soluble reagents (for example, HA instead of β-TCP) can be used. Moreover, the use of monodisperse and fine powder particles is vital to provide an overall fast setting reaction.

Brushite cements are biocompatible and bioresorbable. In contrast to AP cements, they are rapidly resorbed *in vivo* and undergo a rapid decrease in strength (even though strength of the healing bone increases as bone ingrowth occurs). Short setting times, low mechanical strength, and a lack of sufficient fluidity to enable injection through hypodermic needles, prevent brushite cements from use in broader clinical applications. Wet tensile strength of 10 MPa and wet compressive strength of 30 MPa were obtained for brushite cements [45,46]. Pyrophosphate ions, chondroitin 4-sulfate, citrate ions, pyrophosphoric acid and carboxylic acids, such as citric, tartaric and glycolic acids have been used to increase cement setting time and improve the mechanical properties of brushite cements [47,48,49,50,51].

Brushite cements have a fast and linear degradation rate of 0.25 mm/week [51], which might lead to formation of an immature bone. Adding β-TCP granules to the cement paste could solve this problem because they act as bone anchors and encourage formation of a mature bone [51,52]. 

The biocompatibility of brushite based cements has been tested in various experimental applications, compositions and *in vivo* [25,26,53]. Brushite cements are generally well tolerated by the bone and soft tissue environment *in vivo*, such that cement resorption was closely followed by new bone formation. Histological measurements indicated good biocompatibility of brushite cements, with almost complete absence of inflammatory cells [54]. Moreover, bone conduction over an intact cortical surface was obtained with brushite cements, indicating that they may be used in minimally invasive vertical bone augmentation procedures without the need for stabilizing devices.

## 3. Incorporation of Mg, Sr and Zn Ions into TCP Structures

The presence of foreign ions into the structure of synthetic calcium phosphate phases used as starting powders in the formulation of CPCs can alter a series of structural, physico-chemical and biological properties, such as, lattice parameters, crystallinity, solubility in the setting liquid, resorption and bone bonding capability [28,29,55,56]. Under this perspective, a number of research results have been reported so far on the trace elemental incorporation into the synthetic apatites, such as Mg, Sr, Zn, and others.

Among CaP ceramics, β-TCP is greatly biocompatible and resorbable in bone tissue [57,58]. β-TCP implants are as osteoconductive as HA ceramic implants, but simultaneously they are replaced by new bone tissue after some time. Therefore, β-TCP combines the property of biodegradability with that of osteoconductivity, in such a sense that the process taking care of its biodegradation is exactly the process that also induces the formation of new bone. As a result, β-TCP is an adequate ion carrier.

Incorporation of ions into α, β-TCP structures has been experimentally proven through quantitative phase analysis and structural refinement of the powders performed by X-ray diffraction and Rietveld refinement techniques [31,34,56,58,59,60,61,62]. It has been found empirically that extensive ion substitutions can occur in a crystal if the valence of the replacing ion is within one unit of that of the ion being replaced and if the radii of the two ions are similar [63]. Moreover, the accommodation of a foreign ion into the atomic site purely depends on the prevailing conditions in the crystallographic site and also on the nature and size of the ion. Ca, Mg, Zn and Sr fulfil both requirements and these ions can occupy the same site in a crystal, as a result giving solid solution possibilities. The partial substitution of different cations by Ca^2+^ may cause expansion or shrinkage of the β-TCP lattice parameters (a-axis, c-axis and volume) according to the ionic radius of the ions towards Ca^2+^ (0.96 Å).

The incorporation of Mg^2+^ and Zn^2+^ into β-TCP structure was confirmed by a decreasing trend in the lattice parameter values and a contraction of calculated cells volumes. The reason for this contraction in refined cell parameters is the lower ionic radii of Mg (0.72 Å) and Zn (0.745 Å) than Ca ion [59,53,64,65,66]. On the other hand, the incorporation of Sr^2+^ led to an expansion of lattice parameters, due to the greater ionic radius of Sr^2+^ (1.13 Å) in comparison to Ca^2+^ [58,61]. For example, Kannan *et al.* [31] observed a linear expansion of lattice constant values with the increase in the concentration of Sr that was accommodated in the β-TCP structure. Moreover, the results showed that substitution of Sr^2+^ ion in β-TCP structure had greater beneficial influence on the crystallinity. In addition, Bigi *et al.* [27] proved that β-TCP can host up to 80 atom % of Sr with a linear enlargement of the lattice constants, which suggests that Sr incorporation does not provoke a remarkable rearrangement of the unit cell. Regarding the substitution of elements, it has been previously described that β-TCP could be stabilized by the inclusion of small cations without significant alteration in its rhombohedral structure [67].

Concerning chemical properties, it was concluded by the same authors [61,68] that the degree of lattice disturbance by the substituted ions had implications in terms of thermal stability of the β-TCP phase and, therefore, in the easiness and extent of β-TCP → α-TCP phase transformation at higher temperatures (1,450–1,550 °C). Moreover, the lattice strains due to incorporation of larger size cations, such as Sr, favoured the milling process and made the starting Sr-substituted powder more reactive towards the setting liquid in comparison to undoped α-TCP or doped with other cations. Mg has been also reported to have a stabilising role of non-crystalline CaPs, preventing crystallisation into other more stable CaP phases [69].

## 4. Overview of Brushite-Forming Mg-, Zn- and Sr-Substituted Cements Properties

Physico-chemically, CaP surfaces sustain dissolution-reprecipitation cascades as the result of exchanges at a solid-liquid interface in supersaturated conditions. In biological systems, this physico-chemical phenomenon is the result of a multi-component dynamic process involving ions and proteins. In terms of surface reactivity, ionic transfers occur from the solid phase to the aqueous liquid via surface hydration of calcium (Ca), inorganic phosphate species, and possible impurities like carbonate, fluoride, or chloride present in the biomaterial. Under physiological conditions, this dissolution process is highly dependent on the nature of the CaP substrate [70,71,72], and on the composition and supersaturation of the environment *in vitro* [73], or of the implantation site *in vivo* [74,75]. The presence of Mg and carbonate contributes to the formation of a poorly crystallized carbonate apatite that has similar features to the bone mineral phase [76]. In addition, the presence of some additives reduces the *in vitro* and *in vivo* dissolution process, e.g., Mg or Zn in β-TCP [76].

Implanted bone tissues take benefits from the initial setting characteristics of the cements that give, in an acceptable clinical time, a suitable mechanical strength for a shorter tissue functional recovery. It has been shown that ionic substitutions also affect these properties [69].

### 4.1. Setting and Flow Behaviour

Initial setting time of a CPC cement paste and the flow properties are of paramount importance in many applications as *in situ* fracture fixation in orthopaedics, filling root canals and sealing furcation in endodontics and vertebroplasty. CPC should not harden too fast to allow moulding or injection and not harden too slow to permit the surgeon to close the defect shortly after cement placement. Setting times of brushite cements are too fast and the setting can occur in a few seconds [41]. Several factors can control this process by retarding the setting reaction, such as, ionic modification, liquid to powder (L/P) ratio, particle size, particle shape, and additives. Among theses factors, a particular attention is given to Sr, Mg and Zn incorporation into the structure of β-TCP as starting powder. In some of our previous studies [60,68], setting times of 5 ± 0.5 min and 10.0 ± 0.5 min respectively, for Sr- and Mg-substituted brushite-forming cements (L/P = 0.35 mL g^-1^) were determined using the Vicat needle method. Sr enhanced the reactivity of the cement pastes, while Mg tended to delay the setting process in comparison to pure α-TCP cements. This fact was attributed to the stabilizing effect of Mg on the β-TCP structure. Accordingly, the remaining less reactive β-TCP phase present in Mg-substituted cement enabled a longer handling time. Furthermore, in substituted (Sr- and Zn-) β-TCP based cements, a faster setting was also observed for Sr-containing cements in comparison to Zn [61]. On the other hand, additives such as gelling agents can also be used to control the setting reaction. For example, a delay of the setting process was observed in the referred studies [68] when poly(ethylene glycol) (PEG) and hydroxypropylmethylcellulose (HPMC) were used as gelling agents, being less pronounced in the case of PEG, in comparison to HPMC. These agents are hydrophilic polymers that form a network structure with water molecules, which become less free to spontaneously react with inorganic CaPs. On the other hand, the polymeric species might adsorb at the surface of the CaP particles delaying the dissolution/precipitation processes. Consequently, the formed cement network may integrate the polymer structure. This phenomenon is more emphasized when the polymer presents high molecular weight as in the case of HPMC, which could explain the more accentuated decrease of setting time in the presence of this additive in comparison to cement pastes with added PEG.

Rheological properties are crucial in gaining understanding of the fundamentals of the dynamics of flow of injectable CPCs through the delivery system (cannula) and its subsequent interdigitation into the cancellous bone. This knowledge can also help in optimizing the design of the cannula, establishing the optimum time for injection and the optimum viscosity at the time of injection, and minimizing the risk for cement extravasations [42]. Besides, the first initial setting period, within which the cements lose gradually their plasticity, can be assessed by rheological measurements as well.

**Figure 1:**
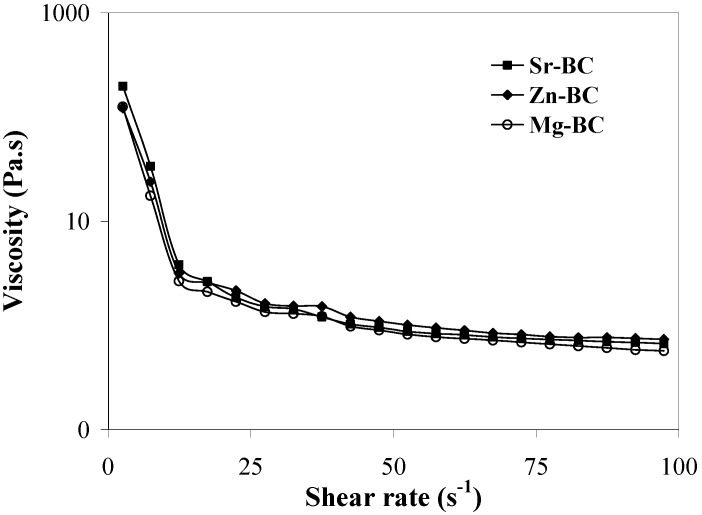
Viscosity versus shear rate curves of the cement pastes prepared with L/P of 0.35 mL g-1. Sr-BC: Sr-substituted bone cement; Zn-BC: Zn-substituted bone cement; Mg-BC: Mg-substituted bone cement.

Bone cements in general are considered viscoelastic materials as they change from having primarily liquid-like properties immediately after mixing to having primarily solid-like properties once cured. As an example, flow curves for Sr-, Mg- and Zn-substituted cement pastes exhibiting similar shear-thinning characteristics are presented in Figure 1.

### 4.2. Injectability and Cohesion

Injectability and cohesion are required for applications with limited accessibility and narrow cavities, and when there is a need for precise placement of the paste to conform to a defect area, such as periodontal bone repair and tooth root canal fillings. However, liquid-phase separation (the so-called filter-pressing effect) provoked by the extrusion pressure applied to the cement paste after a certain injection time has often been observed in commercial formulations. This problem has been the focus of several previous studies [44,49].

A good cohesion of the cement pastes during mixing is essential to avoid the possible occurrence of inflammatory reactions. Cohesion is reached when no disintegration of the cement paste is observed in the fluid, which can be obtained by keeping a high viscosity or using cohesion promoters (e.g., 1% aqueous solution of sodium alginate) and other chemicals [50,77,78,79,80]. Alkhraisat *et al.* [80] found that the combination of carboxylic acids with silica gel was highly efficient in improving cement cohesion, significantly decreasing particle release from the cement surface [81]. Moreover, those cements resulted in shorter final setting time also expected to have better cohesion.

**Figure 2:**
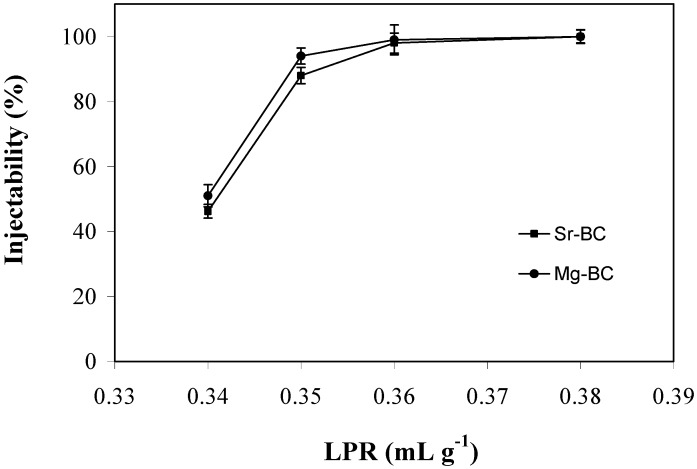
Injectability of the cement pastes as function of L/P, 1.5 min after starting mixing the powder with the setting liquid. Sr-BC: Sr-substituted bone cement; Mg-BC: Mg-substituted bone cement (adapted from [60]).

As aforementioned, there are different factors that can influence injectability and cohesion, such as L/P ratio, particle size and particle size distribution, the injection device, the plastic limit and the use of additives in the mixing liquids [82]. Among them, the factor having the highest influence may be the L/P ratio, as can be observed, for example, by the dependence of the injectability of the cement pastes on the L/P ratio in the range 0.34–0.38 mL g^-1^, 1.5 min after starting mixing the powder with the setting liquid (Figure 2). It can be seen that the injectability increased with increasing L/P ratio, due to the concomitant decrease of the viscosity of the pastes (Figure 1). All the extrusion curves present similar behaviours with the shortest and the longest extrusion plateau being observed for the Sr-, and the Mg-substituted cement pastes, respectively.

### 4.3. Mechanical Strength

Up to now the main criterion used to describe the quality of CPC has been the compressive strength (CS). CPCs are far stiffer than cancellous bone (10–30 MPa), since they are strong enough under compression with values in the range of 10–100 MPa, whereas they possess a low tensile strength (1–10 MPa) [83]. One must bear in mind that the value found for mechanical properties depends, not only on the product, but also on the storage conditions. In particular, the condition of soaking for at least 1 day in water, Ringer’s solution or PBS solution at 37 °C is thought to be relevant for evaluating the suitability of these cements as biomaterials. As shown by Driessens [84], the strength of brushite cements obtained by mixing α-TCP or TTCP, with acidic solutions, depends critically on their storage conditions. It has also been reported in literature that the wet CS values for brushite-forming cements are in the range 1–25 MPa [41]. These mechanical properties may also vary with implantation time, and animal studies have shown that mechanical properties of AP cements tend to increase continually, in contrast to those of brushite cements, which initially decrease and then increase when bone grows [85,86]. This is the result of different porosity and bioresorption between AP and brushite cements. The dimensions of the samples might also be important. Most of authors have adopted the dimensions used in International Standards Organization (ISO) standards for dental cements and for acrylic bone cements: cylinders with a diameter of 6 mm and a height of 12 mm.

Among the product-related factors affecting the strength, the ionic exchange must also be taken into account. To date, Lilley *et al.* [87] have shown that the presence of Mg had a strong effect on cement composition and strength, namely by increasing the proportion of brushite and decreasing the CS. According to these authors [87], Mg could be used to adjust the composition and rate of hydration of the cement. Mg has been also reported to have a stabilising role of non-crystalline CaPs, preventing crystallisation into other more stable CaP phases [69], in good agreement with an observed decrease of the hydrolysis extent of brushite [87]. Klammert *et al.* [88] reported a significant improvement of CS of brushite cement by using Mg-substituted β-TCP. CS of set cements was doubled from 19 MPa to more than 40 MPa after 24 h wet storage. Pina *et al.* [68] showed that Mg-containing brushite-forming cement appeared slightly stronger in comparison to Mg-free cements, due to a more favourable crystalline phase composition. Moreover, another study from the same authors [61] demonstrated that Sr-containing cement specimens exhibited higher CS in comparison to Sr-free cements. In a later report [60], the role of Mg- and Sr-substituted cements relating to CS was evaluated. It was demonstrated that Sr-substituted cements were mechanically stronger (~21 MPa) than with Mg-substituted ones (~19 MPa), after 24 h PBS storage, demonstrating that other benefits can be obtained by properly designing the bone composition. A study by Alkhraisat *et al.* [58] also showed that Sr-substituted β-TCP did not negatively affect the CS of the cements. The values obtained were in the range 5–7 MPa, after 24 h storage in distilled water, at a constant P/L ratio of 3.0 g mL^-1^.

### 4.4. Biological Performance

One of the most appealing characteristics of CPCs is their resorbability *in vivo*. Upon implantation, these materials act as osteoconductive scaffolds but, as time passes, they degrade and are replaced by new bone during the remodelling process [89,90]. The products of the cements’ degradation are Ca^+2^ and PO_4_^3-^ ions, which are easily excreted or recycled by the body. CPCs degradation occurs by the combination of two processes: (i) *In vivo* dissolution, which is strictly related to their composition and particle size [91], and (ii) cell-mediated resorption mainly by osteoclasts. Cell-mediated resorption is advantageous since it mimics the natural process of bone turn-over, in which osteoclasts resorb bone and osteoblasts subsequently secrete bone matrix [92,93]. The bioresorption process of bone substitutes obviously depends on their chemical nature, being closely related with CaPs solubility in aqueous solutions.

Ionic exchange phenomena occurring with CaP bioceramics are associated with reactivity towards bone bonding, i.e., the formation of an interfacial mineralized layer between bioceramics and bone tissue that insures their cohesion. The adverse effect of trace mineral deprivation on bone metabolism in animals has been recognized for many years and is known to be related to specific defects in organic bone matrix synthesis. To date, Mg has acted as a surrogate for Ca in transport and mineralization processes [94,95], but it also exerts a large number of other actions, including enzyme co-factor function and modulation of the action of hormones, growth factors, and cytokines. Mg also has direct effects on the bone formation processes of resorption and mineral aggregation [96]. For example, a recent study [88] showed that Mg-containing brushite-forming cement enabled the osteoblast cells to proliferate and to express the differentiation marker of ALP. Moreover, adding Mg ions to the cement paste can retard the transformation of brushite into HA and thus reduces the possibility of inflammation [42]. Sr-containing brushite cements have been shown to be as good as Sr-free cements in providing a template for cell growth and function [58]. Zn deficiency causes a reduction in osteoblastic activity, collagen and chondroitin sulfate synthesis, and ALP activity [34,98]. Zinc is essential for life and reproduction and is a component of the cell nucleus, mitochondria, cytoplasm, cell membranes, and cell walls [99]. Zinc is a constituent of about 300 enzymes, and Zn ions are located in the catalytic site as well as in the structural site of the enzyme complex [100,101].

**Figure 3:**
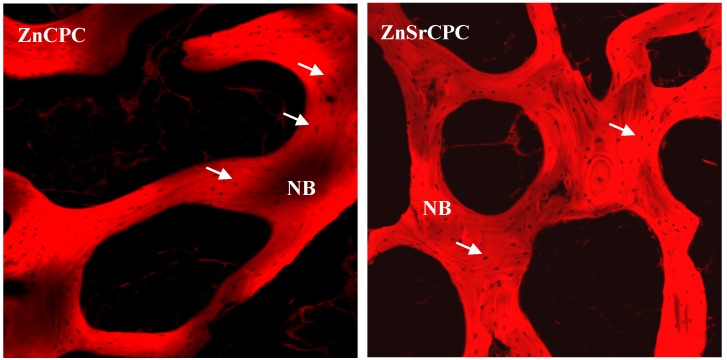
Confocal fluorescent micrographs of histologcal H&E stained sections showing the tissue osteoconductive response to Zn-substituted brushite cements (ZnCPC) and ZnSr-substituted brushite cements (ZnSrCPC), after two months of implantation. NB: new bone; arrows: osteoclast-like cells [102].

Recent studies on Zn- and ZnSr-substituted brushite cements injected into trabecular bone defects in pigs proved that Zn and Sr are good inductors of osteoprogenitor cell proliferation and differentiation [102]. Results indicate that the investigated cements are biocompatible, osteoconductive, and good candidate materials to use as bone substitutes. The pattern of trabecular bone that was formed in the implantation area was qualitatively similar to the adjacent old trabecular bone in the sections’ periphery. Also, results indicated that Sr is a more potent inhibitor of osteoclastic activity than Zn, as much fewer osteoclast-like cells could be found in ZnSr-containing implants (Figure 3), in good agreement with previous studies indicating that Sr-containing ionic cements are more osteoconductive than Zn-containing ionic cements [58,103,104].

Considering the relevance of the above referred ions on bone metabolism in animals it is of paramount importance to grant the required doses in the physiological fluid. In this context, brushite-based cements having higher resorption/solubility rates when compared to apatite cements are advantageous [58,105]. Further, the resorption/solubility of brushite cements can be tailored by doping with silica gel [80]. Moreover, brushite cements have also been proposed as drug deliver systems. Alkhraisat *et al.* [104] investigated the effect of Sr substitution on drug release from brushite cements. They used Sr-substituted CPC loaded with doxycycline hyclate (DOXY-h) and found that Sr-substitution in cements increases the cement specific surface area, improving DOXY-h adsorption. All the studies referred above demonstrate the versatility of substituted brushite-forming cements for clinical applications.

## 5. Conclusions

Despite few studies in the literature regarding Mg, Zn and Sr substituted brushite-forming bone cements, the results obtained so far revealed that these cements are good candidates for applications in repair of bony and periodontal defects, owing their relevant properties (*i.e.*, setting time, injectability, mechanical strength) for clinical application, especially the Sr-containing cements that exhibited an overall better performance.

There remains a need for controlled, optimization and prospective studies of ionic-substituted brushite cements regarding specific clinical applications, such as, vertebroplasty and kyphoplasty, by studying and defining the needs in terms of cement properties and driving the research towards the adequate solutions. Future developments will enable the commercialization of better and more differentiated products that should improve the clinical outcome and hence the patient life quality.
